# Preparation of hybrid particles of Ag nanoparticles and eggshell calcium carbonate and their antimicrobial efficiency against beef-extracted bacteria

**DOI:** 10.1098/rsos.221197

**Published:** 2023-05-24

**Authors:** Moe Ei Ei Zin, Pornpimol Moolkaew, Tiraporn Junyusen, Wimonlak Sutapun

**Affiliations:** ^1^ School of Polymer Engineering, Institute of Engineering, Suranaree University of Technology, Nakhon Ratchasima 30000, Thailand; ^2^ Research Centre for Biocomposite Materials for Medical and Agricultural and Food Industry, Suranaree University of Technology, Nakhon Ratchasima 30000, Thailand; ^3^ School of Agricultural Engineering, Institute of Engineering, Suranaree University of Technology, Nakhon Ratchasima 30000, Thailand

**Keywords:** antimicrobial agent, silver nanoparticles, eggshell calcium carbonate, silver loaded eggshell calcium carbonate, food packaging, co-precipitation

## Abstract

In this study, hybrid particles of AgNPs-loaded eggshell calcium carbonate (AgNPs/eCaCO_3_) were prepared by co-precipitating the eggshell in the presence of freshly prepared AgNPs with a particle size of 10–30 nm. The hybrid particles were comparatively precipitated at 25°C and 35°C using poly (sodium 4-styrenesulfonate) as a polyelectrolyte. The AgNPs/eCaCO_3_ particles prepared at 25°C had a spherical morphology with a mean diameter of 3.56 µm, and Brunauer–Emmett–Teller (BET) surface area of 85.08 m^2^ g^−1^. On the other hand, the particles prepared at 35°C had a broader size distribution with a mean diameter of 3.19 µm, and a BET surface area of 79.25 m^2^ g^−1^. AgNPs-loaded commercial calcium carbonate particles (AgNPs/CaCO_3_) comparatively prepared at 35°C were perfectly spherical with a mean diameter of 5.61 µm. At preparing temperature of 25°C, the hybrid particles contain AgNPs of 0.78 wt% for AgNPs/eCaCO_3_ and 3.20 wt% for AgNPs/CaCO_3_. The AgNPs/eCaCO_3_ and AgNPs/CaCO_3_ particles exhibited the same efficiency against bacteria extracted from beef with an average inhibition zone diameter of 7–10 mm according to the modified Kirby–Bauer disc diffusion assay depending on their concentration and beef source. Freshly prepared silver colloids showed comparatively poorer antimicrobial efficiency.

## 1. Introduction

Several types of metal and metal oxide nanoparticles such as Ag, Au, TiO_2_, CuO and ZnO, have gained attention owing to their antimicrobial efficiency and high surface area per volume. Ag nanoparticles (AgNPs) are less expensive than Au and TiO_2_. The TiO_2_ nanoparticles are effective antifungal agents against fluconazole-resistant strains and are suitable for photocatalytic activity. Ag, CuO and ZnO nanoparticles show antibacterial activity against Gram-negative and Gram-positive bacteria. However, Ag nanoparticles (AgNPs) are the most popular antimicrobial agents because of their high activity against drug-resistant bacteria, high stability and non-toxicity [1]. Moreover, AgNPs are highly effective antimicrobial agents against a wide spectrum of Gram-negative and Gram-positive bacteria; fungi such as *Aspergillus niger* and *Candida albicans*; and viruses including HIV-1, hepatitis B virus, respiratory syncytial virus, herpes simplex virus type 1 and monkeypox virus [2–5]. The cytotoxicity of AgNPs is derived from the nanoparticles themselves and Ag^+^ released from oxidative dissolution. In aqueous environments, AgNPs suspensions are oxidized in the presence of oxygen and protons, thereby releasing Ag^+^ via surface oxidative dissolution [2]. The oxidative dissolution reaction is shown in equation (1.1) [6]. The release rate of Ag^+^ depends on the size, shape, surface properties, capping agent and colloidal state of AgNPs [7].

1.1 $${\mathrm{Ag}}^{0}(s)+(1/4)O_{2}(\mathrm{aq})+H^{+}(\mathrm{aq})↔{\mathrm{Ag}}^{+}(\mathrm{aq})+(1/2)H_{2}O.$$

**Figure 1.**
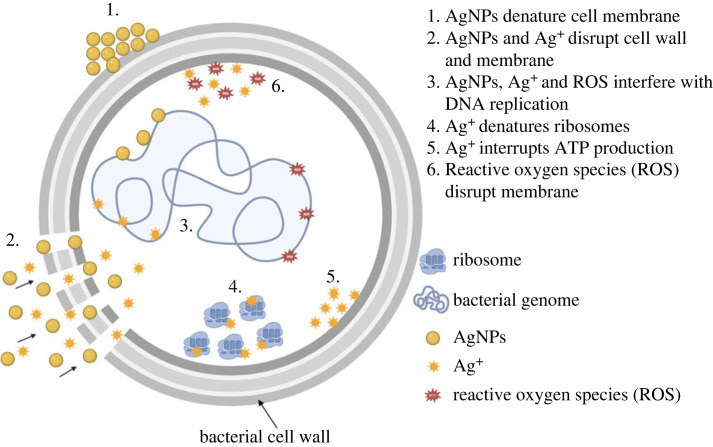
The antibacterial mechanism of silver nanoparticles (AgNPs) [8].

Although the mechanisms underlying the activity of AgNPs against bacteria have not yet been fully clarified, various antibacterial mechanisms have been proposed. Yin *et al*. proposed the following possible mechanisms: (i) disruption of the cell wall and membrane by Ag^+^, (ii) denaturation of ribosomes by Ag^+^, (iii) interruption of adenosine triphosphate (ATP) production by Ag^+^, (iv) disruption of the cell membrane by reactive oxygen species (ROS), (v) interference of DNA replication by AgNPs, Ag^+^ and ROS, (vi) denaturation of the cell membrane by AgNPs, and (vii) perforation of the membrane by AgNPs [8]. A diagram of the antibacterial mechanisms of AgNPs drawn using *BioRender* is shown in figure 1. Durán *et al*. reported that ROS could be a principal agent in the induction of cell membrane disruption and deoxyribonucleic acid (DNA) modification. The interaction of Ag^+^ with sulfur and phosphorus, which are important DNA compounds, can cause problems in DNA replication and cell reproduction, or even result in the termination of microorganisms. Furthermore, the synthesis of proteins can be inhibited by Ag^+^, causing denaturation of ribosomes in the cytoplasm [9].

The cytotoxicity of AgNPs is highly dependent on several factors such as size, shape, surface chemistry, stability, surface charge, capping agent, pH, ionic strength and degree of agglomeration. Size-dependent cytotoxicity of AgNPs has been demonstrated in many studies. Smaller particles have a larger specific surface area, which can expose a large number of atoms on the surface for redox, photochemical and biochemical reactions, as well as physico-chemical interactions with cells [5]. In addition, the presence of ligands, divalent cations and macromolecules also plays a key role on cytotoxicity [2,5,10].

Silver nanoparticles could be prepared via physical, chemical, green synthetic and biological methods. The physical methods result in low yields. The chemical reduction of a silver salt in aqueous media is a common method for producing AgNPs with reducing compounds such as sodium borohydride (NaBH_4_) [11,12], trisodium citrate (Na_3_C_6_H_5_O_7_) [13–15], glucose (C_6_H_12_O_6_), hydrazine (N_2_H_4_) and ascorbate (C_6_H_7_O_6_) [5]. Trisodium citrate was commonly used as a reducing and stabilizing agent for silver nitrate to produce AgNPs [16]. The citrate reduction of silver colloids serves the dual role of the reductant and stabilizer. The silver particles prepared by the citrate reduction method were relatively large sized (50–100 nm) with well-defined facets [14]. AgNPs can be also green synthesized from plant-extracted chemicals and biologically obtained from yeast extracts [17–19]. Preferred particle size and distribution, and morphology of the AgNPs can be obtained via the chemical techniques with suitable parameters. By utilizing low-toxic chemicals as reducing agent and stabilizing agent in the reduction chemical method, the harmful effects on the environment and hazardous by-products will be reduced.

Owing to growing concerns about antibiotics resistance, incorporating AgNPs into different matrices/supporters/carriers has emerged as an effective way to produce materials with high antimicrobial efficiency. AgNPs incorporated into polymer matrices are currently a popular type of nanocomposite with excellent antimicrobial properties. Antimicrobial polymer composites can be applied in food packaging containers [20], food packaging films [21], wound dressing materials [22,23] and other antimicrobial applications [24,25] owing to their outstanding antimicrobial activity. One of the main challenges in producing this type of antimicrobial is the synthesis of stable nanoparticles, as their antimicrobial effectiveness greatly depends on their size, size distribution and agglomeration state [26]. Notably, the most common application problem involves the agglomeration and diffusion of these nanoparticles into a polymer matrix, which reduces antibacterial activity. To prevent agglomeration and maintain the long-lasting antimicrobial activity of AgNPs, porous micro CaCO_3_ particles in vaterite polymorph have been used as carriers for metallic nanoparticles [16,27,28]. Lei *et al*. synthesized vaterite CaCO_3_ in the presence of poly (sodium 4-styrene-sulfonate) of a specific size and morphology [29]. Using porous micro CaCO_3_ particles as carriers or supporters, the antimicrobial activity of the active AgNPs can be prolonged owing to reduced agglomeration of the nanoparticles as well as limiting contact of AgNPs with the human body [16]. Moreover, chemical and physical techniques for synthesizing CaCO_3_ containing AgNPs as hybrid particles must be made greener using either low-toxicity chemicals or raw materials from bioresources for sustainability.

Chicken eggshells from household waste are extensively used as a replacement for mineral calcium carbonate in several applications such as plastic and elastomer fillers, heavy metal absorbents, dye removal bio-calcium supplements, concrete replacement and biodiesel oil catalysts [30]. Eggshells are readily available with a high CaCO_3_ content (94 wt%), in the form of calcite polymorph [31,32] with 7000 and 17 000 pores [33,34] and the smallest pore size of 3–13 µm [33,34]. In addition, chicken eggshells contain other inorganic compounds including magnesium carbonate (1%), calcium phosphate (1%) and organic matter (4%) [35]. Eggshell waste can be used as an alternative and green precursor for hybrid particles of AgNPs and CaCO_3_.

The number of patents on eggshell applications has increased exponentially in the last 7 years, from 150 in 2014 to 250 in 2020, emphasizing biotechnological applications involving biomedical, chemical, engineering and environmental technology [36]. One of the biotechnological applications of eggshell calcium carbonate is as a hybrid particle. The ceramic–ceramic and ceramic–metal hybrid particles exploit the advantages of the synergistic function of the compositions of the hybrid particles. For example, TiO_2_/SiO_2_, a ceramic–ceramic hybrid particle, can be applied as a functional filler for elastomers and possesses the advantages of photocatalytic properties and antimicrobial biomaterials by TiO_2_, as well as high thermal stability and excellent mechanical strength by SiO_2_ [37]. For ceramic–metal hybrid particles, AgNPs/CaCO_3_ could be used as an antimicrobial agent, with CaCO_3_ acting as an AgNPs carrier and AgNPs as an active antimicrobial agent. The CaCO_3_ carrier also improves the distribution and distributive properties of AgNPs [16,28].

The patent filed by Yang *et al*. disclosed a method to produce hybrid particles of AgNPs and CaCO_3_ using eggshells as a template. Eggshells were pulverized and loaded with Ag^+^, followed by heating at 400–600°C. The resultant hybrid particles can be used for various applications including catalysis, tissue engineering, coatings and the production of antibacterial agents, pigments and ceramics [38]. Nevertheless, the proposed method consumes a large amount of energy while heating up to 600°C. Hassan *et al*. prepared eggshell CaCO_3_ platelets using a combination of mechanochemical and sonochemical techniques. The obtained CaCO_3_ platelets had particle sizes, Brunauer–Emmett–Teller (BET) surface areas and pore volumes of 10 nm, 43.69 m^2^ g^−1^ [32] and 0.164 cm^3^ g^−1^ [39], respectively. Apalangya *et al*. employed a mechanochemical milling technique to deposit spherical AgNPs with a diameter of 5–20 nm onto the surface of micrometre-sized ground eggshells with crystal polymorphs of calcite [28]. They also mentioned that macromolecular proteins and functional groups in the eggshells, such as hydroxyl, carboxyl and amino functional groups could stabilize and immobilize AgNPs onto the surfaces of eggshell particles. However, the morphology of the ground eggshells obtained using the mechanochemical milling technique is platelet-shaped, resulting in the anisotropic properties for the hybrid particles. Zapotoczny *et al*. prepared hybrid particles of AgNPs and CaCO_3_, for sustained release applications, using a calcium nitrate and sodium carbonate solution for co-precipitation in the presence of freshly prepared silver colloids [16]. The particle size of the AgNPs was 10–30 nm. However, crystal polymorphs of the co-precipitated CaCO_3_ have not yet been reported.

In addition to the above-mentioned techniques involving the preparation of ground eggshell powders, hybrid particles of AgNPs and eggshell CaCO_3_, and AgNPs and mineral CaCO_3_, this article proposes a two-step method using the green raw material of bio-eggshell waste to prepare hybrid particles of AgNPs and CaCO_3_ as antimicrobial agents. The two-step method comprising silver colloids preparation and silver colloids/eggshell CaCO_3_ co-precipitation. Using the two-step method, the specific particle size, size distribution and morphology of the hybrid particles of AgNPs and eggshell CaCO_3_ were chemically controllable with a higher BET specific surface area and pore volume.

In this study, AgNPs were used as an active agent against the growth of bacteria contaminating fresh beef. Due to their highly effective antimicrobial activity against a wide spectrum of Gram-negative and Gram-positive bacteria, biocompatibility and low production cost. Veterite calcium carbonate was prepared from eggshell and functioned as the AgNPs carrier due to its porous structure, isotropic property and non-toxicity. To prepare the hybrid particles of AgNPs and vaterite calcium carbonate (AgNPs/eCaCO_3_), AgNPs were freshly synthesized by a chemical reduction method using trisodium citrate, a low toxicity chemical, and then deposited *in situ* into micro-sized eggshell calcium carbonate particles during precipitation. In addition, hybrid particles of AgNPs loaded with commercial calcium carbonate (AgNPs/CaCO_3_) were comparatively prepared. Furthermore, the effect of precipitation temperature on the particles size and size distribution of AgNPs/eCaCO_3_ was investigated. The hybrid particles were characterized using field emission scanning electron microscopy (FESEM), transmission electron microscopy (TEM) and energy dispersive X-ray spectroscopy (EDS, EDX) to monitor particle size and size distribution; particle morphology; and silver content, respectively. Dynamic light scattering (DLS) technique was also employed to study the hydrodynamic radius of the AgNPs. BET analysis was used to determine the specific surface area, pore volume and pore diameter of the particles. Finally, the antimicrobial activities of silver colloids, AgNPs/eCaCO_3_ particles, AgNPs/CaCO_3_ particles and precipitated eCaCO_3_ particles against beef-extracted bacteria were compared using a modified Kirby–Bauer disc diffusion assay. Soon, AgNPs/eCaCO_3_ particles will be incorporated into a biodegradable poly (lactic acid) using the electrospinning method for antimicrobial food packaging. Using eggshell as a precursor for preparing Ca(NO_3_)_2_ in precipitating vaterite calcium carbonate for carrying AgNPs has not been previously reported. Effect of the eggshell precipitation temperature has not been studied, as well. This study offers the novel co-precipitation technique for preparing hybrid particles of AgNPs and micrometre-sized vaterite calcium carbonate using bio-green source chicken eggshell with the determined mixing step and temperature to control the size of AgNPs, and morphology and size of vaterite calcium carbonate. For the hybrid particles aiming as antimicrobial agents in beef packaging, AgNPs function as active antimicrobial agents and vaterite calcium carbonate as carriers or supporters.

## 2. Materials and methods

### 2.1. Materials

Chicken eggshells (ES) were obtained from household waste, and commercial calcium carbonate particles were obtained from Sand and Soil Industry Co., Ltd. Silver nitrate (≥99.0%, ACS reagent), sodium carbonate (≥99.5%, ACS reagent), sodium citrate tribasic dihydrate (trisodium citrate) (≥99.0%, ACS reagent) and poly (sodium 4-styrenesulphonate) (PSS, average M_w_∼70 000 g mole^−1^) were obtained from Sigma Aldrich. Nitric acid (65%, AR grade) was purchased from ANaPURE. Plate count agar (PCA) and peptone were purchased from HiMedia Laboratories Pvt. Ltd and Sisco Research Laboratories Pvt. Ltd, respectively. Unpacked fresh beef and vacuum-packed fresh beef (MAX BEEF) were purchased from a local fresh market and a Home-Fresh Mart supermarket, respectively.

### 2.2. Preparation of eggshell powder

Household waste eggshells were washed with tap water and boiled in a rice cooker at 100°C for 4 h. Subsequently, eggshell membranes were peeled off. The eggshells were crushed into small pieces and dried at room temperature for 24 h. To obtain fine eggshells pieces, a grinding machine (Retsch, SR300) was used to reduce the initial size of the dried eggshells. To remove all the remaining biomacromolecules, such as the eggshell membrane, the eggshell powder was washed with water at least five times and then dried in an oven at 60°C for 24 h. Ball milling with different ball sizes was used for further size reduction. After 24 h of ball milling, the eggshell powder was sieved through No. 500 and No. 450 sieves, to obtain particle size of 25–32 µm.

### 2.3. Preparation of calcium nitrate solution

To prepare the calcium nitrate solution, 102 g of eggshell powder (ESP) was dissolved in 1000 ml of 2 M nitric acid solution. After the ESP was completely dissolved, titration was performed with a 0.5 M sodium hydroxide solution using phenolphthalein as an indicator to determine the exact molarity of the calcium nitrate solution, after which the pH of the solution was adjusted to neutral using a 0.50 M sodium hydroxide solution. To obtain a molarity equivalent to that of sodium carbonate solution, the calcium nitrate solution was diluted to 0.03 M concentration.

For comparison, 100 g of commercial calcium carbonate was dissolved in a 2 M nitric acid solution. To get 0.03 M calcium nitrate solution from commercial calcium carbonate, the same procedure mentioned in the previous paragraph was performed.

### 2.4. Preparation of silver nanoparticles deposited on eggshell CaCO_3_ and commercial calcium carbonate

Hybrid particles of silver nanoparticles (AgNPs) deposited on eggshell CaCO_3_ and commercial calcium carbonate were prepared using a co*-*precipitation method, as described by Zapotoczny *et al*. [16]. First, to prepare the silver colloids, 45 mg of silver nitrate was dissolved in 250 ml of deionized (DI) water, and then 5 ml of 1 w/v% trisodium citrate was added. The reaction mixture was placed in an ultrasonic bath and heated at 70–72°C for 60 min.

At the same time, PSS was dissolved in DI water to get a 4.8 g l^−1^ PSS solution, and a 0.03 M sodium carbonate solution was prepared. Then, 20 ml of the freshly prepared silver colloids was simultaneously mixed with 50 ml of 0.03 M Ca(NO_3_)_2_ solution prepared from either eggshell calcium carbonate or commercial calcium carbonate and 50 ml of 0.03 M Na_2_CO_3_ solution with the addition of 15 ml of PSS (4.8 g l^−1^). The mixture was then sonicated for 5 min at 25°C. The obtained particles were washed with DI water and centrifuged at 4000 r.p.m. for 5 min to remove the excess silver. The washing process was repeated thrice, and the obtained particles were dried under vacuum at 40°C for 24 h. AgNPs/eCaCO_3_ and precipitated eCaCO_3_ were also prepared at a sonication temperature of 35°C via the same procedure.

### 2.5. Sample characterization

The particle morphology and size of AgNPs, precipitated eCaCO_3_, AgNPs/eCaCO_3_ and AgNPs/CaCO_3_ were determined using a field emission scanning electron microscope (FESEM, JEOL JSM 7800F) with an accelerating voltage of 3.0 kV. Prior to observation, each sample was coated using a gold sputter coater (Neo-Coater, MP-19020NCTR) for 2 min. The diameter of the particles was measured from the SEM micrographs using an analysis software (ImageJ) and calculated by randomly selecting 200 particles. Matlab R2022a was used to obtain the mean diameter, size distribution and histogram.

Moreover, the morphology and elemental composition of the AgNPs, precipitated eCaCO_3_, AgNPs/eCaCO_3_ and AgNPs/CaCO_3_ were investigated using a transmission electron microscope (TEM, Thermo Scientific Talos F200X) coupled with energy dispersive X-ray spectroscopy (EDX). The morphology and size distribution of the nanoparticles from the TEM micrographs were recorded digitally, and the elemental composition of the particles was determined by EDS in the mapping mode using Velox software. Samples for TEM were prepared by depositing a drop of each sample in distilled water on a carbon-coated standard copper grid (200 meshes) and allowed to dry before investigation.

The crystal polymorphs of the ground eggshells, precipitated eCaCO_3_ and hybrid particles were determined by an X-ray diffraction analyser (XRD, Bruker D8 ADVANCE). The study was carried out in a 2*θ* range of 5–80° with a voltage of 40 kV, current of 40 mA and a Cu K*α* (1.5606 Å) radiation source.

To study the decomposition temperature of ground eggshells and precipitated eCaCO_3_ prepared at 35°C, a thermogravimetric analyser (TGA, Mettler Toledo, TGA/DSC1) was employed. The samples were heated from 35 to 1000°C at a heating rate of 10°C min^−1^ under nitrogen atmosphere.

The hydrodynamic particle size and polydispersity index (PDI) of silver colloids and AgNPs, was determined by dynamic light scattering analyser (DLS, Malvern Instruments Zetasizer Nano ZS). The measurements were carried out in triplicate at 25°C with laser wavelength of 633 nm, beam back-scattering angle of 173° and DI water's refractive index of 1.330.

The BET surface area and porosity of all particles were measured using a BET analyser (Micromeritics 3Flex). All samples were degassed at 40°C for 24 h under vacuum before examination.

### 2.6. Antimicrobial test

To examine the antimicrobial efficiency of the hybrid silver nanoparticles/calcium carbonate particles on beef-extracted bacteria, a modified Kirby–Bauer disc diffusion assay was performed as follows. Bacteria were extracted from two types of beef, unpacked beef available at a local market and vacuum-packed beef available at a supermarket (‘MAX BEEF’ brand). The beef loaf was cut into small pieces and kept at room temperature for 18 h. The powdered forms of precipitated eCaCO_3_, AgNPs/eCaCO_3_ and AgNPs/CaCO_3_ were dispersed in sterile DI water and kept at 4°C overnight. Then, 2.35 g of plate count agar (PCA) powder was completely dissolved in 100 ml sterile DI water using a hot plate. Next, the PCA solution was sterilized, and then poured into sterile Petri dishes after the solution and the dishes were cool down to 45–50°C. Approximately 25 g of spoiled beef was blended with 225 ml of 0.10% sterile peptone water using a Stomacher laboratory blender for 6 min. The obtained beef extract was smeared onto plate count agar using a sterile cotton swab. Four sterile circular Whatman filter paper discs (6 mm diameter) were placed onto the smeared agar plate and impregnated with 5 µl of precipitated eCaCO_3_, freshly prepared silver colloids (silver colloids at 0 h), AgNPs/eCaCO_3_ and AgNPs/CaCO_3_. The test was performed at particle concentrations of 500, 250 and 125 mg ml^−1^. The plates were incubated at 37°C for 24 h. After that, each inhibition zone diameter was monitored, and digital images of the antimicrobial zone were taken. The diameters were averaged from the three measurements. The antimicrobial test was performed in triplicate and the average diameter of the inhibition zone with standard deviation was calculated.

## 3. Results and discussion

### 3.1. Characteristic of silver nanoparticles

**Figure 2.**
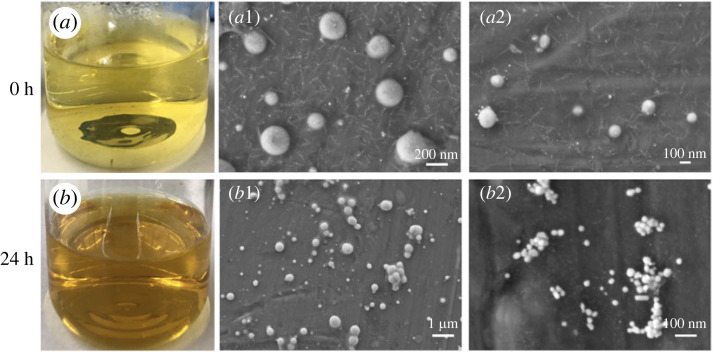
The image of freshly prepared silver colloids (silver colloids at 0 h) (*a*) and SEM micrographs of AgNPs obtained from the silver colloids at 0 h with a magnification of 50k× (*a*1) and 50k× (*a*2). The image of silver colloids at 24 h (*b*) and SEM micrographs of AgNPs obtained from the silver colloids at 24 h with a magnification of 10k× (*b*1) and 100k× (*b*2).

The optical micrographs in figure 2*a,b* show images of the silver colloids over time. Freshly prepared silver colloids are clear and light-yellow, resulting in both large and exceedingly small silver nanoparticles (AgNPs) of approximately 100–200 nm and 1 nm, respectively, as shown in figure 2*a*1,*a*2. However, after being left at room temperature for 24 h, the silver colloids became dark brown (figure 2*b*) because the small nanoparticles became larger. The AgNPs began to grow over time, as confirmed by the SEM micrographs shown in figure 2*b*1,*b*2. Agglomeration can also be observed in the SEM micrograph shown in figure 2*b*2.

**Figure 3.**
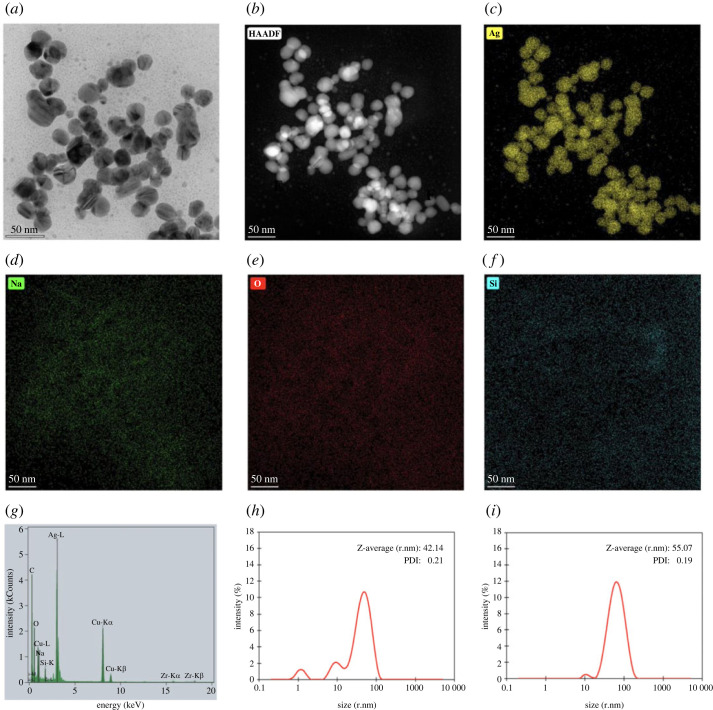
TEM micrograph (*a*), high-angle annular dark-field imaging (HAADF) (*b*), Silver (Ag) EDS image mapping (*c*), Sodium (Na) EDS image mapping (*d*), Oxygen (O) EDS image mapping (*e*), Silicon (Si) EDS image mapping (*f*) and EDX spectrum (*g*) of AgNPs obtained from freshly prepared silver colloids, and the hydrodynamic size distribution curves of (*h*) and AgNPs obtained from (*i*) freshly prepared silver colloids.

TEM micrographs, EDS mapping images and EDX spectrum of AgNPs obtained from freshly prepared silver colloids are shown in figure 3. The TEM micrographs show the spherical morphology of AgNPs, as observed from SEM in figure 2, and the average size of the individual particles was approximately 10–30 nm. Particles with diameters of less than 10 nm were observed as small dots. The EDS image mapping and EDX spectrum in figure 3*c–g* illustrate that the particles were mainly composed of silver. Large-sized (50–100 nm) silver crystallites were found using trisodium citrate as a reducing and stabilizing agent [14]. Arif *et al*. also studied the synthesis of silver nanoparticles in the presence of trisodium citrate, resulting in diameters of approximately 40 nm AgNPs [40]. According to the smaller particle size and lesser agglomeration, the freshly prepared silver colloids were further used to prepare AgNPs-loaded calcium carbonate particles as antimicrobial agents.

The freshly prepared silver colloids contained the Z-average (r.nm) of 42.1 nm and PDI of 0.2, as observed from figure 3*h*. The hydrodynamic size distribution curve of freshly prepared silver colloids, shown in figure 3*h*, shows three peaks at 50.0, 10.3 and 1.2 nm. This distribution curve corresponded well with their TEM micrograph in figure 3*a*, showing three different sizes of the particles. In addition, the Z-average size (r.nm) and PDI of AgNPs, obtained from the freshly prepared silver colloids, were 55.1 and 0.2, with two peaks at 69.6 and 11.0 nm as illustrated by the hydrodynamic size distribution curve in figure 3*i*. The 1 nm AgNPs might be extracted out during the centrifugal separation; as a result, there are two particle size ranges observed from the distribution curve. Ranoszek-Soliwoda *et al*. [41] prepared AgNPs at 100°C using trisodium citrate as a capping agent. They reported hydrodynamic size (d.nm) of the prepared AgNPs in ranges of 9 ± 2 nm and 58 ± 20 nm, with PDI of 0.5, and the particle size of the AgNPs observed from scanning transmission electron microscopy (STEM) in ranges of 5–45 nm and larger than 100 nm.

### 3.2. Characteristic of precipitated eggshell calcium carbonate (eCaCO_3_) particles

**Figure 4.**
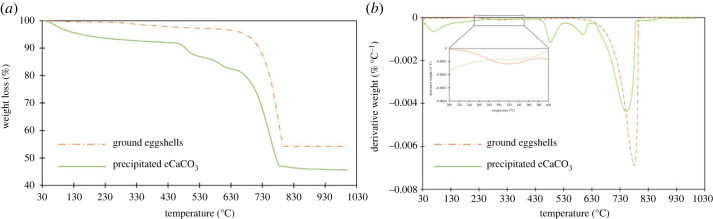
TGA curves (*a*) and DTGA curves (*b*) of ground eggshells and precipitate eCaCO_3_ prepared at 35°C.

The TGA and differential thermogravimetric analysis (DTGA) curves of the ground eggshells and precipitated eCaCO_3_ prepared at 35°C are shown in figure 4*a*,*b*. Ground eggshells exhibited three thermal transitions of mass loss. The first thermal decomposition occurred in the temperature range of 35–110°C due to the evaporation of physically adsorbed water. The second transition in the range of 240–350°C caused by thermal decomposition of the eggshell matrix left in the ground eggshell. The TGA curve shows overall mass loss of 1.9% from water evaporation and eggshell matrix decomposition. Castro *et al*. also reported that natural eggshells showed a mass loss of 1.9% between 30 and 400°C [42]. The third mass loss occurred between 400 and 800°C, with a DTGA peak at 783°C and a mass loss of 45.8%. This loss is related to the CO_2_ released from CaCO_3_ decomposition. Decarbonization of chicken eggshells has been reported to occur in the temperature range of 600–850°C [43,44].

In the case of precipitated eCaCO_3_ prepared at 35°C, the TGA and DTGA curves show four regions of thermal decomposition with an overall weight loss of approximately 46.2%. The first weight loss at 35–180°C was due to the evaporation of physically adsorbed water from CaCO_3_. The second and third decompositions in the temperature range of 430–520°C and 550–650°C, were derived from the thermal degradation of PSS in precipitated eCaCO_3_. These two thermal decomposition ranges were also reported by Bahrom *et al*. [45]. They reported that the thermal decomposition of precipitated CaCO_3_ using PSS as an organic additive at 430–520°C and 550–650°C was due to the decomposition of PSS. The fourth thermal decomposition at 790°C corresponded to the decarbonization of vaterite CaCO_3_. It could be concluded that there was no organic matter left in the precipitated eCaCO_3_, and vaterite polymorph precipitated from eggshell absorbed more water than calcite polymorph of eggshell.

**Figure 5.**
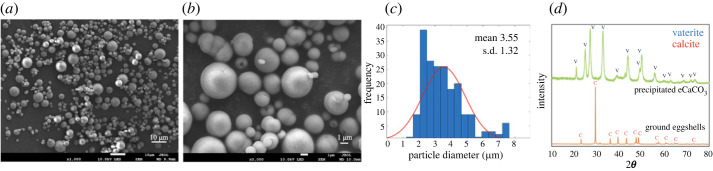
SEM micrographs with magnification of 1k× (*a*) and 5k× (*b*), and a particle size distribution curve (*c*) of precipitated eCaCO_3_ particles prepared at 35°C, and XRD patterns of the precipitated eCaCO_3_ particles and ground eggshells (*d*).

**Figure 6.**
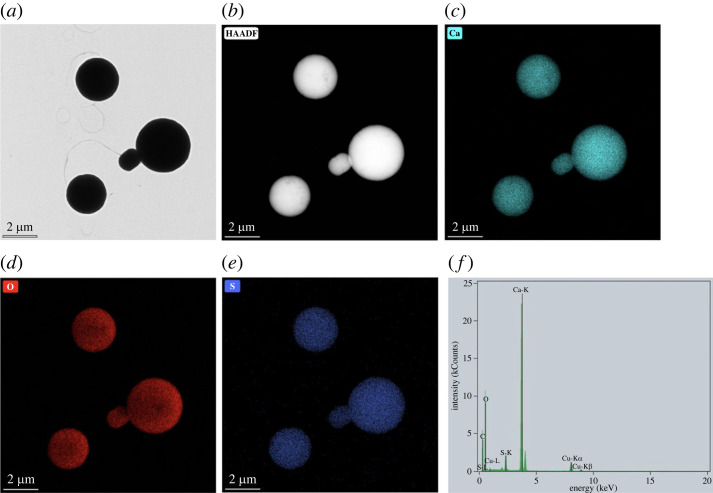
TEM micrograph (*a*), high-angle annular dark-field imaging (HAADF) (*b*), calcium (Ca) EDS image mapping (*c*), oxygen (O) EDS image mapping (*d*), sulfur (S) EDS image mapping (*e*) and EDX spectrum (*f*) of precipitated eCaCO_3_ particles prepared at 35°C.

SEM micrographs in figure 5*a*–*c* show spherical morphology and a broad particle size distribution of precipitated eCaCO_3_ particles prepared at 35°C, with a mean diameter of 3.55 ± 1.32 µm. The mechanism of CaCO_3_ formation involves the creation of nanometre-sized crystallites in the first stage which later aggregate to form micrometre-sized superstructures [16]. According to the XRD pattern in figure 5*d*, crystal polymorphs of the precipitated eCaCO_3_ prepared at 35°C was vaterite, whereas that of the ground eggshells was calcite. Sutapun *et al*. also reported that ground eggshells were mainly composed of a calcite crystal structure [43]. The vaterite crystal structure is formed when CaCO_3_ is precipitating in the presence of poly (sodium 4-styrenesulfonate) (PSS) as a polyelectrolyte. Lei *et al*. studied precipitating vaterite CaCO_3_ from 0.5 M CaCl_2_ and 0.5 M Na_2_CO_3_ and 70 000 g mole^−1^ and 1 g l^−1^ PSS at pH 10 and the obtained CaCO_3_ particles were vaterite polymorph with micrometre-sized spherical shapes. It was deduced that PSS initiated vaterite nucleation through the binding of calcium ions to anionic sulfonate groups, and stabilized the vaterite during crystal growth [29]. Azarian also found that the crystal structure of precipitated calcium carbonate from calcium nitrate and sodium carbonate in the presence of polyelectrolytes was vaterite with a spherical morphology [46]. The TEM micrographs in figure 6*a*,*b* also show a broad particle size distribution. The EDS image mapping and EDX spectrum in figure 6*c*–*f* illustrated that the particles were rich in calcium carbonate.

### 3.3. Characteristic of silver nanoparticles ***/*** eggshell calcium carbonate (AgNPs***/***eCaCO_3_) particles

**Figure 7.**
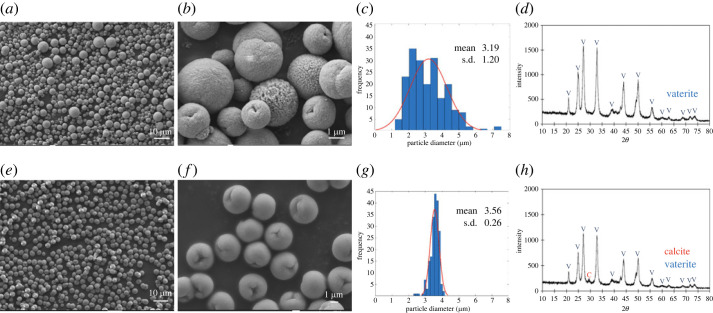
SEM micrographs with magnification of 1k× (*a*) and 10k× (*b*), a particle size distribution curve (*c*) and an XRD pattern (*d*) of AgNPs/eCaCO_3_ particles prepared at 35°C. SEM micrographs with magnification of 1k× (*e*) and 5k× (*f*), a particle size distribution curve (*g*) and an XRD pattern (*h*) of AgNPs/eCaCO_3_ particles prepared at 25°C.

Figure 7*a*–*d* show the particle morphology, histogram plot of particle diameter versus frequency and XRD spectrum for AgNPs/eCaCO_3_ particles prepared at 35°C. The spherical morphology of AgNPs/eCaCO_3_ particles with a particle size distribution ranging from 1.44 to 7.38 µm and a mean diameter of 3.19 ± 1.20 µm was obtained. Figure 7*e*–*h* show the morphology, histogram plot of particle diameter versus frequency and XRD spectrum for AgNPs/eCaCO_3_ particles prepared at 25°C. The XRD patterns in figure 7*d*,*h* confirm that the particles prepared at 25°C and 35°C were mainly composed of vaterite crystal structures. The obtained AgNPs/eCaCO_3_ particles also showed a spherical morphology with a narrow size distribution ranging from 2.39 to 4.15 µm and a mean diameter of 3.56 ± 0.26 µm. One of the factors affecting particle size and distribution of precipitated CaCO_3_ is crystallization temperature. At high temperature, the crystallization is governed by kinetic factors. Mathawa *et al*. studied crystallization of CaCO_3_ at 25 and 80°C using polyacrylic acid (PAA) as a stabilizing agent. They stated that when the crystallization was carried out at high temperature, the dissolution of CaCO_3_ from the surface of growing particles might have occurred, and the secondary crystallization might then be formed. This secondary crystallization resulted in smaller CaCO_3_ crystals compared with the larger primary crystals [47].

At an ambient temperature of 25°C, the precipitated eCaCO_3_ particles crystallized homogeneously with a narrow particle size distribution, and they did not form perfect spherical structure like precipitated commercial calcium carbonate particles as shown in figure 9. This is because other compositions of chicken eggshells such as magnesium carbonate (1%) and calcium phosphate (1%) might disturb the crystallization [35].

**Figure 8.**
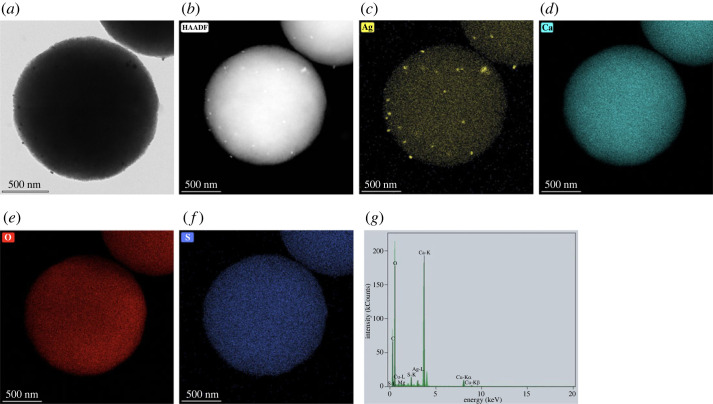
TEM micrograph (*a*), high-angle annular dark-field imaging (HAADF) (*b*), silver (Ag) EDS image mapping (*c*), calcium (Ca) EDS image mapping (*d*), oxygen (O) EDS image mapping (*e*), sulfur (S) EDS image mapping (*f*) and EDX spectrum (*g*) of AgNPs/eCaCO_3_ particles prepared at 25°C.

TEM micrographs, EDS image mapping and EDX spectrum of AgNPs/eCaCO_3_ prepared at 25°C in figure 8 show that AgNPs are attached to the eggshell calcium carbonate particles. The TEM micrographs and EDS image mapping in figure 8*a*–*c* confirm the presence of AgNPs on both the surface and inside of eCaCO_3_. AgNPs are attached to the whole particle of calcium carbonate resulting in silver content of 0.78 wt% confirmed through EDS mapping element analysis. According to the EDX spectrum shown in figure 8*g*, the particles were composed solely of silver and calcium carbonate and no other impurities were observed. So, AgNPs/eCaCO_3_ particles prepared at 25°C were further used as antimicrobial agents against food-related bacteria due to their smaller size and narrow distribution.

### 3.4. Characteristic of silver nanoparticles ***/*** commercial calcium carbonate (AgNPs***/***CaCO_3_) particles

**Figure 9.**
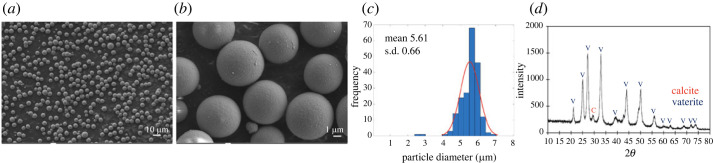
SEM micrographs with magnification of 500× (*a*) and 5k× (*b*), a particle size distribution curve (*c*) and an XRD pattern (*d*) for AgNPs/CaCO_3_ particles prepared at 25°C.

**Figure 10.**
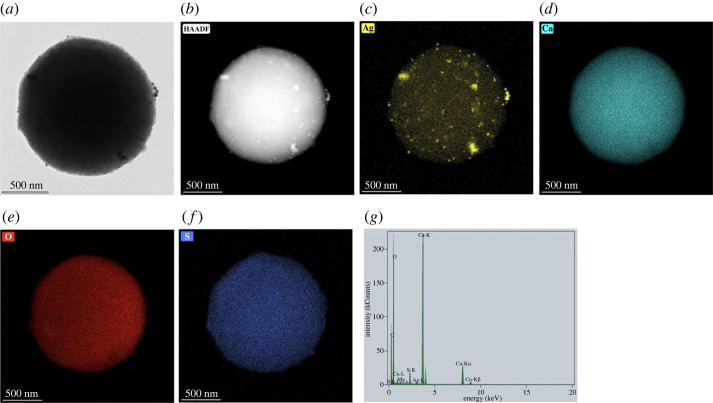
TEM micrograph (*a*), high-angle annular dark-field imaging (HAADF) (*b*), silver (Ag) EDS image mapping (*c*), calcium (Ca) EDS image mapping (*d*), oxygen (O) EDS image mapping (*e*), sulfur (S) EDS image mapping (*f*) and EDX spectrum (*g*) of AgNPs/CaCO_3_ particles.

The morphology, size distribution and XRD pattern of the AgNPs/CaCO_3_ particles are shown in figure 9*a*–*d*. According to the SEM micrographs, the AgNPs/CaCO_3_ particles prepared at 25°C were spherical microparticles with a particle size distribution ranging from 4.15 to 6.95 µm and a mean diameter of 5.61 ± 0.66 µm (figure 9*c*). The particles are dominant in the vaterite crystal structure, as shown in the XRD pattern in figure 9*d*. The spherical shape of the precipitated commercial calcium carbonate was loaded with AgNPs on both the surface and inside of the calcium carbonate particles as shown by the TEM micrographs and EDS image mapping in figure 10*a*–*c* with a silver content of 3.20 wt%. The EDX spectrum in figure 10*g* confirms that the particles contain only silver and calcium carbonate. Zapotoczny *et al*. prepared AgNPs loaded calcium carbonate particles at 25°C using calcium nitrate and sodium carbonate, resulting in a particle size of 2 µm, in approximation [16]. In this study, AgNPs/eCaCO_3_ and AgNPs/CaCO_3_ prepared at 25°C using eggshell and commercial calcium carbonate as a precursor had a mean particle size of 3.56 ± 0.26 µm and 5.61 ± 0.66 µm, respectively.

The specific surface area, total pore volume and mean pore diameter of precipitated eCaCO_3_ and AgNPs/eCaCO_3_ prepared at 35°C were remarkably similar, as shown in table 1. The specific area of the hybrid particles of AgNPs and CaCO_3_ prepared at 25°C was higher than that prepared at 35°C. At co-precipitation temperature of 25°C, the specific area of AgNPs/eCaCO_3_ was approximately the same as that of AgNPs/CaCO_3_. However, the mean pore diameter of the hybrid particles of AgNPs and CaCO_3_ prepared at 25°C was slightly smaller than that of the particles prepared at 35°C. All particles mentioned in table 2 have the same total pore volume (*V*) of around 0.07 m^3^ g^−1^ and a mean pore diameter (d_p_) in a range of 3.3–3.8 nm.

**Table 1.** Silver content, BET specific surface area (*S*_BET_), total pore volume (*V*), and mean pore diameter (*d*_p_) of precipitated eCaCO_3_, AgNPs/eCaCO_3_ and AgNPs/CaCO_3._

particles	silver content (wt%)	*S*_BET_ (m^2^ g^−1^)	*V* (m^3^ g^−1^)	*d*_p_ (nm)
precipitated eCaCO_3_ prepared at 35°C	—	79.77	0.07	3.72
AgNPs/eCaCO_3_ prepared at 35°C	—	79.25	0.07	3.78
AgNPs/eCaCO_3_ prepared at 25°C	0.78	85.08	0.07	3.26
AgNPs/CaCO_3_ prepared at 25°C	3.20	85.85	0.07	3.39

**Table 2.** Inhibition zone diameter against extracted bacteria from unpacked and vacuum-packed beef using precipitated eCaCO_3_, freshly prepared silver colloids, AgNPs/eCaCO_3_ and AgNPs/CaCO_3_ as antimicrobial agents.

samples	inhibition zone diameter (mm)					
	unpacked beef-extracted bacteria			vacuum-packed beef extracted bacteria		
	500 mg ml^−1^	250 mg ml^−1^	125 mg ml^−1^	500 mg ml^−1^	250 mg ml^−1^	125 mg ml^−1^
precipitated eCaCO_3_	no effect	no effect	no effect	no effect	no effect	no effect
freshly prepared silver colloids	6.99 ± 0.12	6.77 ± 0.12	6.57 ± 0.19	7.38 ± 0.50	7.17 ± 0.58	7.13 ± 0.33
AgNPs/eCaCO_3_	8.76 ± 0.26	8.24 ± 0.67	8.98 ± 0.47	10.52 ± 1.06	8.56 ± 0.22	7.44 ± 0.47
AgNPs/CaCO_3_	8.97 ± 0.49	8.09 ± 0.31	8.27 ± 0.31	10.16 ± 0.80	7.89 ± 0.81	7.45 ± 0.38

### 3.5. Antimicrobial efficiency

**Figure 11.**
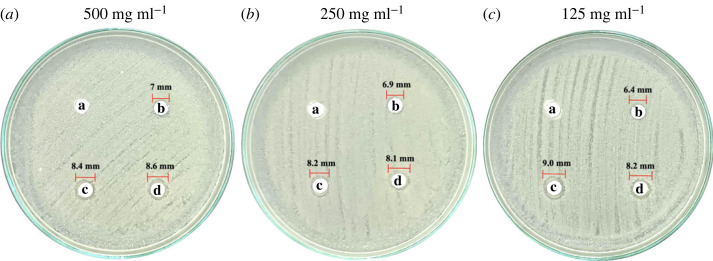
Antimicrobial activity against bacteria extracted from unpacked raw beef from a local fresh market. The discs were impregnated with precipitated eCaCO_3_ (a), freshly prepared silver colloids (b), AgNPs/eCaCO_3_ (c), and AgNPs/CaCO_3_ (d), at concentrations of 500 mg ml^−1^ (*a*), 250 mg ml^−1^ (*b*), and 125 mg ml^−1^ (*c*).

**Figure 12.**
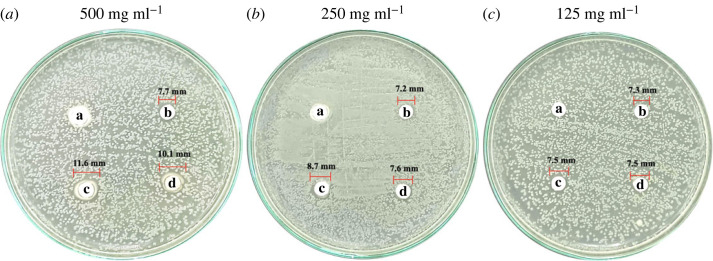
Antimicrobial activity against bacteria extracted from vacuum-packed beef (‘MAX BEEF’ brand). The discs were impregnated with precipitated eCaCO_3_ (a), freshly prepared silver colloids (b), AgNPs/eCaCO_3_ (c), and AgNPs/CaCO_3_ (d), at concentrations of 500 mg ml^−1^ (*a*), 250 mg ml^−1^ (*b*) and 125 mg ml^−1^ (*c*).

As shown in figures 11*a*–*c* and 12*a*–*c*, precipitated eCaCO_3_ (disc 'a') showed no inhibitory effect against bacteria extracted from unpacked beef purchased from the fresh market and vacuum-packed beef of ‘MAX BEEF’ brand. The freshly prepared silver colloids showed slight antimicrobial activity against the bacteria extracted from the unpacked beef and the vacuum-packed beef with inhibition zone diameters of approximately 7 mm, as observed from disc ‘b’ in figures 11*a*–*c* and 12*a*–*c*, respectively. The antimicrobial efficiency of the silver colloids was lower than that of AgNPs/eCaCO_3_ and AgNPs/CaCO_3_. This might be caused by the agglomeration of the silver colloids within the disc, in which the dissolution of Ag^0^ or Ag^+^ was slower than that of the hybrid particles of AgNPs/eCaCO_3_ and AgNPs/CaCO_3_ [26]. Lee *et al*. investigated the antimicrobial activity of AgNPs prepared at different molar ratios using the Kirby–Bauer disc diffusion assay and demonstrated a good inhibitory effect against *Escherichia coli* (*E. coli*) and *Staphylococcus aureus* [48]. The antimicrobial efficiency depends on the particle size, particle size and concentration [18,19], and the degree of agglomeration of nanoparticles [26]. Therefore, the superior antimicrobial activity of AgNPs/eCaCO_3_ and AgNPs/CaCO_3_ particles compared with that of the silver colloid was attributed to the reduced agglomeration of AgNPs, giving rise to a better stabilized and sustained release of AgNPs from the eCaCO_3_ and CaCO_3_ carriers [28]. It was reported that 20 nm AgNPs showed antimicrobial against *Escherichia coli* at MIC (minimum inhibitory concentration) of 0.621 mg ml^−1^ [49]. In addition, 5 nm and 10 nm AgNPs exhibited MIC at 0.625 mg ml^−1^ and 1.35 mg ml^−1^, respectively, against *Staphylococcus aureus* [50].

In addition, AgNPs/eCaCO_3_ and AgNPs/CaCO_3_ showed concentration-independent antimicrobial activity against bacteria extracted from unpacked beef. The diameters of the inhibition zones at 125, 250, and 500 mg ml^−1^ were not significantly different, with an average diameter of 8–9 mm. This suggested that the optimum concentration of AgNPs/eCaCO_3_ and AgNPs/CaCO_3_ for inhibiting bacteria growth was 125 mg ml^−1^. By contrast, AgNPs/eCaCO_3_ and AgNPs/CaCO_3_ showed concentration-dependent antimicrobial activity against bacteria extracted from vacuum-packed beef. The average inhibition diameter tended to increase with increasing the hybrid particle concentration.

The Laboratory of Cell-Based Assay and Innovation (CBAI), Suranaree University of Technology, reported that the extract from the unpacked beef contained two types of Gram-negative and one type of Gram-positive bacteria, whereas the extract from the vacuum-packed beef contained one type of Gram-negative and one type of Gram-positive bacteria. The lesser types of extracted bacteria from vacuum-packed beef might be the main effect of concentration-dependent antimicrobial activity against the bacteria of the hybrid particles. However, it could be concluded that the antimicrobial efficiencies of AgNPs/eCaCO_3_ and AgNPs/CaCO_3_ against bacteria extracted from unpacked and vacuum-packed beef were comparable.

For the case of AgNPs/CaCO_3_, Zapotoczny *et al*. studied the antimicrobial activity of AgNPs/CaCO_3_ microparticles against *Staphylococcus* and *Candida* species and reported that the AgNPs/CaCO_3_ showed inhibitory effects for those species [16]. Apalangya *et al*. reported that AgNPs/eggshell particles exhibited superior antimicrobial activity against *Escherichia coli* compared with that of AgNPs [28]. The mentioned reports were in good agreement with the inhibitory effect of the hybrid particles of AgNPs/eCaCO_3_ and AgNPs against bacteria extracted from beef, as shown in table 2. In addition, Wang *et al*. mentioned that *Pseudomonas fragi*, *Myroides phaeus* and *Brochothrix thermosphacta* were bacteria found in beef [51], and it was reported that *E. coli* was found in raw beef, as well [52].

The summary of the average inhibition zone diameter with standard deviation obtained from precipitated eCaCO_3_, freshly prepared silver colloids, AgNPs/eCaCO_3_ and AgNPs/CaCO_3_, against extracted bacteria from the unpacked and vacuum-packed beef versus testing concentrations are shown in table 2.

## 4. Conclusion

Spherical AgNPs with an average size of 10–30 nm were successfully prepared by reducing an AgNO_3_ aqueous solution at 70°C in the presence of trisodium citrate as a reducing agent and stabilizer. The Z-average (r.nm) and PDI are 42.1 nm and 0.2, respectively for freshly prepared silver colloids, and 55.1 and 0.2, respectively for AgNPs. Novel hybrid antimicrobial microspherical particles of AgNPs/eCaCO_3_ and AgNPs/CaCO_3_ were successfully prepared via aqueous precipitation in the presence of freshly prepared silver colloids using poly (sodium 4-styrenesulfonate) as a polyelectrolyte. TEM, EDS and EDX confirmed that AgNPs were incorporated into the spherical precipitated CaCO_3_ from eggshells and commercial calcium carbonate. The crystal polymorph of the precipitated eCaCO_3_ was vaterite. The hybrid AgNPs/eCaCO_3_ particles prepared at 25°C had a spherical morphology and a narrow size distribution, with a mean diameter of 3.56 ± 0.26 µm. By contrast, AgNPs/eCaCO_3_ prepared at 35°C showed a broader size distribution with a mean diameter of 3.19 ± 1.20 µm. In addition, 0.78 wt% and 3.20 wt% AgNPs were deposited onto and inside the microspherical particles of precipitated eggshells and commercial calcium carbonate, respectively. AgNPs/CaCO_3_ particles prepared at 25°C also had a spherical morphology and a narrow size distribution with a mean diameter of 5.61 ± 0.66 µm. Therefore, eggshells and commercial calcium carbonate can be applied as carriers or supporters for the nanoparticles.

Antimicrobial test showed that both AgNPs/eCaCO_3_ and AgNPs/CaCO_3_ particles exhibited the same antimicrobial activity. The hybrid microspherical AgNPs/eCaCO_3_ and AgNPs/CaCO_3_ particles inhibited the growth of bacteria extracted from both unpacked and vacuum-packed beef. However, AgNPs/eCaCO_3_ and AgNPs/CaCO_3_ showed concentration-dependent antimicrobial activity against bacteria extracted from vacuum-packed beef.

## Data Availability

The supplements supporting this article are available from the Dryad Digital Repository: https://doi.org/10.5281/zenodo.7080849 [53]. The datasets supporting this article are available from the Dryad Digital Repository: https://doi.org/10.5061/dryad.kd51c5b8w [54]. The data are provided in electronic supplementary material [55].
